# Relationship between iodine nutritional status and low handgrip strength in a Northwestern Chinese cohort: construction of a predictive nomogram model

**DOI:** 10.3389/fnut.2026.1793869

**Published:** 2026-04-14

**Authors:** Xiao Liu, Tao Zhang, Songbo Fu, Donghu Zhen, Jingfang Liu, Haixia Qi, Chengxu Ma, Zhongyan Shan, Lihua Ma

**Affiliations:** 1 Department of Endocrinology, The First Hospital of Lanzhou University, Lanzhou, China; 2 School of Nursing, Lanzhou University, Lanzhou, China; 3 The 940th Hospital of Chinese People’s Liberation Army, Lanzhou, China; 4 Department of Endocrinology and Metabolic Diseases, The First Hospital of China Medical University, Shenyang, China

**Keywords:** determinants, low grip strength, predictive nomogram, restricted cubic spline analysis, urinary iodine concentration

## Abstract

**Background:**

Grip strength is a key marker for sarcopenia screening, yet the influence of iodine nutritional status on low grip strength (LGS) remains poorly understood.

**Methods:**

This cross-sectional study analyzed 810 community-dwelling adults in Lanzhou, China, randomly assigned to training (*n* = 568) and validation (*n* = 242) cohorts. Logistic regression analyses identified factors associated with LGS. A nomogram integrating urinary iodine concentration (UIC), systolic blood pressure (SP), and routine clinical variables was constructed. Model performance was evaluated using ROC curves, Hosmer-Lemeshow test, and Brier scores. Restricted cubic spline (RCS) analysis assessed the dose–response relationship between UIC and LGS, with subgroup analyses by thyroid function.

**Results:**

LGS prevalence was 18.8% in the training cohort. Multivariate analysis showed female sex (OR = 0.34), greater height (OR = 0.94), and excessive iodine (OR = 0.36) were inversely associated with LGS, whereas age (60–74 years: OR = 2.20; ≥75 years: OR = 9.73), smoking (OR = 3.20), and SP (OR = 1.02) correlated with higher LGS odds. The nomogram demonstrated moderate discrimination (AUC: 0.723 training, 0.662 validation) with good calibration (Brier scores: 0.134 training, 0.147 validation; H-L *p* > 0.05). RCS analysis revealed a U-shaped association between UIC and LGS in unadjusted analyses, with the inverse associations most evident among euthyroid participants.

**Conclusion:**

The nomogram provides a practical, objective tool for early identification of individuals at high probability of LGS. Moderate-to-high UIC is associated with LGS odds, particularly in euthyroid adults. This model may guide targeted screening and interventions in community settings. In the cross-sectional design, causal inferences cannot be drawn; Further research is warranted to elucidate these complex relationships.

## 1 Introduction

Sarcopenia is a geriatric syndrome defined by progressive loss of skeletal muscle mass (SMM), strength, and somatic function, which links to conditions such as type 2 diabetes and cognitive dysfunction (1, 2). Grip strength serves as a primary clinical measure for sarcopenia due to its convenience and validity. Research has identified strong correlations between grip strength and depressive symptoms, debilitation, and all-cause mortality (3–6). Specifically, each 1 kg decline in grip strength correlates with a 1.7% increase in heart disease risk (HR = 1.017/1 kg decrease, 95% CI: 0.865–0.976) (7). Despite established links between grip strength and cardiac prognosis, existing studies often focus on single factors and are concentrated in developed urban centers. Evidence regarding low grip strength (LGS) in less developed regions, particularly the association between grip strength and iodine status in the Northwest Territories, remains limited. This study aims to construct a prediction model for LGS and examine the dose–response relationship between LGS and urinary iodine concentration (UIC) using restricted cubic spline (RCS) analysis. This aims to provide a clinical tool for identifying populations with a high probability of LGS, thereby informing early screening strategies.

## 2 Participants and methods

### 2.1 Study population

This study utilized data from the China 10-Year Epidemiological Follow-up Project on Thyroid, Iodine Nutrition, and Diabetes in Gansu Province (July 2024 to April 2025). A multistage stratified sampling method was employed to select 958 permanent community residents eligible for the baseline survey. The inclusion criteria were as follows: (1) age ≥ 18 years; (2) local residency for ≥ 5 years; (3) no missing data on key variables (demographic information, grip strength, laboratory test results). The exclusion criteria were: (1) pregnancy; (2) severe visual or auditory impairments, or communication difficulties; (3) individuals who had consumed seaweed/kelp within the past 3 days, were currently taking iodine-containing medications, or had undergone contrast agent examinations within the past 3 months. Participants with missing data exceeding 20% for key variables (age, sex, grip strength, and UIC) were excluded. A final cohort of 810 participants was included in the analysis.

**Figure 1:**
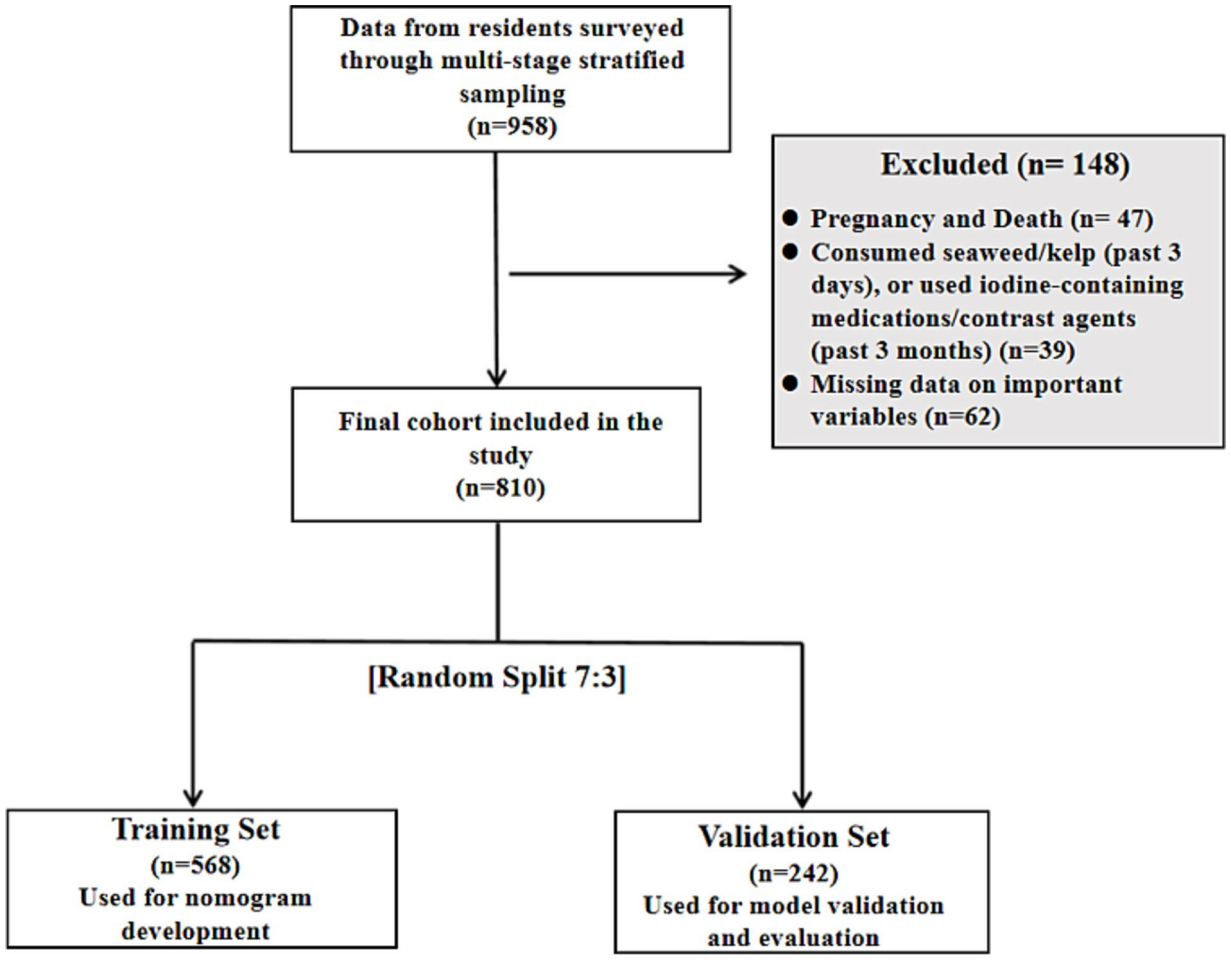
Flowchart of participant selection.

Age stratification was informed by conventional epidemiological categories (8): ≤44 years (young), 45–59 years (middle-aged), 60–74 years (young elderly), and ≥75 years (elderly). UIC was categorized using two distinct approaches. For the multivariable logistic regression analysis, UIC was classified into three groups based on the China Iodine Nutrition and Thyroid Disease Survey criteria (9): iodine deficiency (<100 μg/L), iodine sufficiency (100–299 μg/L), and excess iodine (≥300 μg/L). To better characterize the relationship between UIC and LGS, we also employed a four-category classification for descriptive purposes: iodine deficiency (<100 μg/L), iodine sufficiency (100–299 μg/L), mild iodine excess (300–499 μg/L), and moderate to severe iodine excess (≥500 μg/L). Thyroid function classification was based on TSH, FT3, and FT4 levels. Grip strength was classified into low grip strength (LGS) and normal grip strength groups using the criteria of the Asian Working Group for Sarcopenia (AWGS) (10): <28 kg for males and <18 kg for females. The analytical sample was randomly divided into a training set (*n* = 568) and a validation set (*n* = 242) at a 7:3 ratio using a computerized random number generator. The training set was used for nomogram development, and the validation set was employed for model validation and evaluation (Figure 1).

### 2.2 Methods

(1) For the questionnaire survey, trained and qualified investigators contacted participants via telephone to schedule appointments for them to attend a designated location for the physical examination. The investigators used a standardized guide to explain the procedure for completing the questionnaire. For participants unable to complete the survey physically, the investigator read the questionnaire content aloud, avoiding suggestive questioning, and the investigator recorded the participant’s responses verbatim.

(2) The physical examination included measurements of height, weight, waist, blood pressure, and grip strength. Grip strength was assessed using a Baseline® Hydraulic Grip Strength Gauge (Model 12-0286, USA). Participants were seated with their feet flat on the ground, knees flexed at 90°, shoulder joints retracted, elbows flexed at 90°, forearms in a neutral position, and wrists extended at an angle of 0°-30°. The gauge was held steady, and arm swinging was prohibited during measurements. Participants were instructed to grip the dynamometer firmly and apply maximum force for 2–3 s before releasing. Three measurements were performed for each hand; the highest value for each was recorded, and their average was used as the final grip strength.

(3) Laboratory tests, fasting venous blood and urine samples were collected on the day of the survey. Fasting blood glucose, lipids, uric acid, HbA1c, and thyroid function parameters were measured using standard protocols. The UIC was measured using inductively coupled plasma mass spectrometry (ICP-MS) from Agilent Technologies in the United States.

### 2.3 Statistical analysis

Data were double-entered using EpiData 3.1. Statistical analyses were performed using SPSS 27.0 and R 4.4.2. Normally distributed continuous variables were expressed as mean ± standard deviation (SD) and compared using the independent sample t-test. Non-normally distributed continuous variables were presented as medians with interquartile ranges (IQR), and comparisons between groups were performed using the Mann–Whitney *U* test. Categorical variables were described as frequencies and proportions, with group comparisons conducted using the Chi-square test. To identify predictors of LGS, univariate and multivariate logistic regression analyses were used to explore the relevant factors for the occurrence of LGS, and multicollinearity was assessed using the variance inflation factor (VIF). Variables were entered into a multivariable logistic regression model to calculate adjusted odds ratios (ORs) and 95% CIs. The nomogram prediction model was constructed. The dose–response relationship between continuous UIC and LGS was evaluated using restricted cubic spline (RCS) analysis and validated through subgroup analysis based on thyroid function. The model was developed using the “rms” package in R. The “p ROC” software package was used to plot receiver operating characteristic (ROC) curves to evaluate the model’s predictive performance, and to calculate the area under the curve (AUC) to compare the predictive accuracy of different models. Model performance was further assessed by plotting calibration curves and conducting decision curve analysis (DCA). A two-sided *p <* 0.05 was considered statistically significant.

The study was approved by the Ethics Committee of the First Hospital of Lanzhou University (Approval No. LDYYLL2024-404). Informed consent was obtained from all participants.

## 3 Results

### 3.1 Comparison of baseline information for the training set

A total of 568 study subjects were included in the training set, and the prevalence of LGS was 18.8% (107/568), with an 18.6% prevalence in males and a 20.5% prevalence in females. Univariate analysis revealed that the differences between the LGS and normal grip strength groups were statistically significant for the following variables: age group, smoking status, height, FT4, and the UIC group (*p <* 0.05, Table 1).

**Table 1:** Comparison of the baseline data of patients in the low grip strength and normal grip strength groups.

Variable	Total (*n*)	Non LGS group (*n* = 461)	LGS group (*n* = 107)	*X*^2^/*Z*	*p*-value
Sex, *n* (%)	568			0.022	0.883
Male	285	232 (50.33)	53 (49.53)		
Female	283	229 (49.67)	54 (50.47)		
Age group, *n* (%)				32.744	<0.001*
1 (≤44)	276	236 (51.19)	40 (37.38)		
2 (45–59)	180	152 (32.97)	28 (26.17)		
3 (60–74)	96	67 (14.53)	29 (27.10)		
4 (≥75)	16	6 (1.30)	10 (9.35)		
Smoking, *n* (%)				11.392	0.003*
No smoking	422	341 (73.97)	81 (75.70)		
<20sticks/day	35	22 (4.77)	13 (12.15)		
>20sticks/day	111	98 (21.26)	13 (12.15)		
Alcohol, *n* (%)				3.008	0.557
No alcohol	236	186 (40.35)	50 (46.73)		
>12times/year	181	149 (32.32)	32 (29.91)		
>1time/month	94	77 (16.70)	17 (15.89)		
>1 time/week	50	44 (9.54)	6 (5.61)		
>1 time/day	7	5 (1.08)	2 (1.87)		
Thyroid Function, *n* (%)				0.071	0.789
Normal	435	352 (76.36)	83 (77.57)		
Abnormal	133	109 (23.64)	24 (22.43)		
UIC group, *n* (%)				6.197	0.045*
Iodine deficiency	129	98 (21.26)	31 (28.97)		
Iodine sufficiency	360	292 (63.34)	68 (63.55)		
Excess iodine	79	71 (15.40)	8 (7.48)		
Height (cm)		167.00 (162.00, 173.00)	165.00 (159.50, 171.00)	−2.543	0.011*
Weight (kg)		66.00 (58.00, 76.00)	64.00 (56.25, 73.25)	−1.324	0.185
BMI (kg/m^2^)		23.70 (21.70, 26.00)	23.70 (21.55, 25.80)	−0.185	0.853
HR (bpm)		77.00 (70.00, 86.00)	76.00 (69.00, 82.00)	−1.515	0.130
SP (mmHg)		122.00 (113.00, 133.00)	124.00 (114.00, 137.00)	−1.612	0.107
DP (mmHg)		77.00 (71.00, 85.00)	78.00 (70.00, 85.00)	−0.194	0.846
FBG (mmol/L)		5.60 (5.30, 6.00)	5.70 (5.20, 6.10)	−0.269	0.788
OGTT2h (mmol/L)		7.30 (6.50, 8.50)	7.80 (6.60, 9.50)	−1.370	0.171
HbA1c (%)		5.58 (5.36, 5.81)	5.64 (5.39, 5.99)	−1.769	0.077
WC (cm)		86.00 (77.50, 92.00)	87.00 (80.00, 92.50)	−1.190	0.234
TRIG (mmol/L)		1.49 (1.00, 2.16)	1,47 (1.11, 2.18)	−0.341	0.733
TCHOL (mmol/L)		4.47 (3.90, 5.12)	4.67 (3.91, 5.20)	−1.389	0.165
LDL-CH (mmol/L)		2.39 (1.92, 2.96)	2.57 (1.90, 2.96)	−0.556	0.578
HDL-CH (mmol/L)		1.23 (1.02, 1.48)	1.24 (1.04, 1.52)	−0.732	0.464
TSH (μIU/mL)		2.61 (1.72, 3.93)	2.76 (1.90, 3.81)	−0.766	0.443
UA (mmol/L)		338.00 (279.00, 413.00)	337.00 (283.00, 389.00)	−0.913	0.361
FT3 (pmol/L)		5.21 (4.82, 5.68)	5.10 (4.74, 5.53)	−1.564	0.118
FT4 (pmol/L)		16.41 (15.10, 17.86)	16.09 (14.59, 17.16)	−2.183	0.029*
UIC (μg/L)		166.72 (108.85, 236.17)	139.26 (88.53, 210.80)	−2.715	0.007*

*P < 0.05.

### 3.2 Association between UIC and LGS

**Figure 2:**
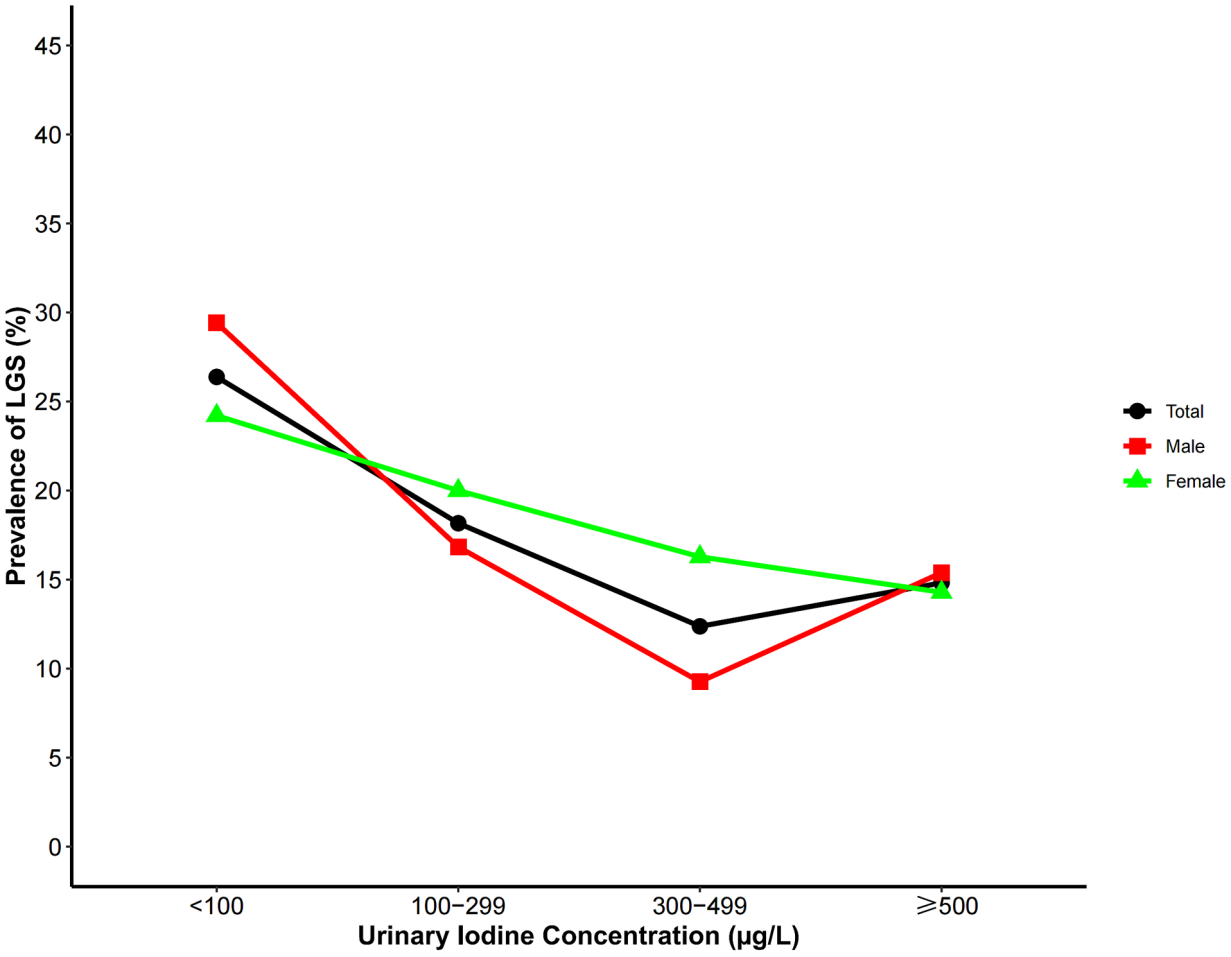
LGS prevalence across UIC levels.

**Figure 3:**
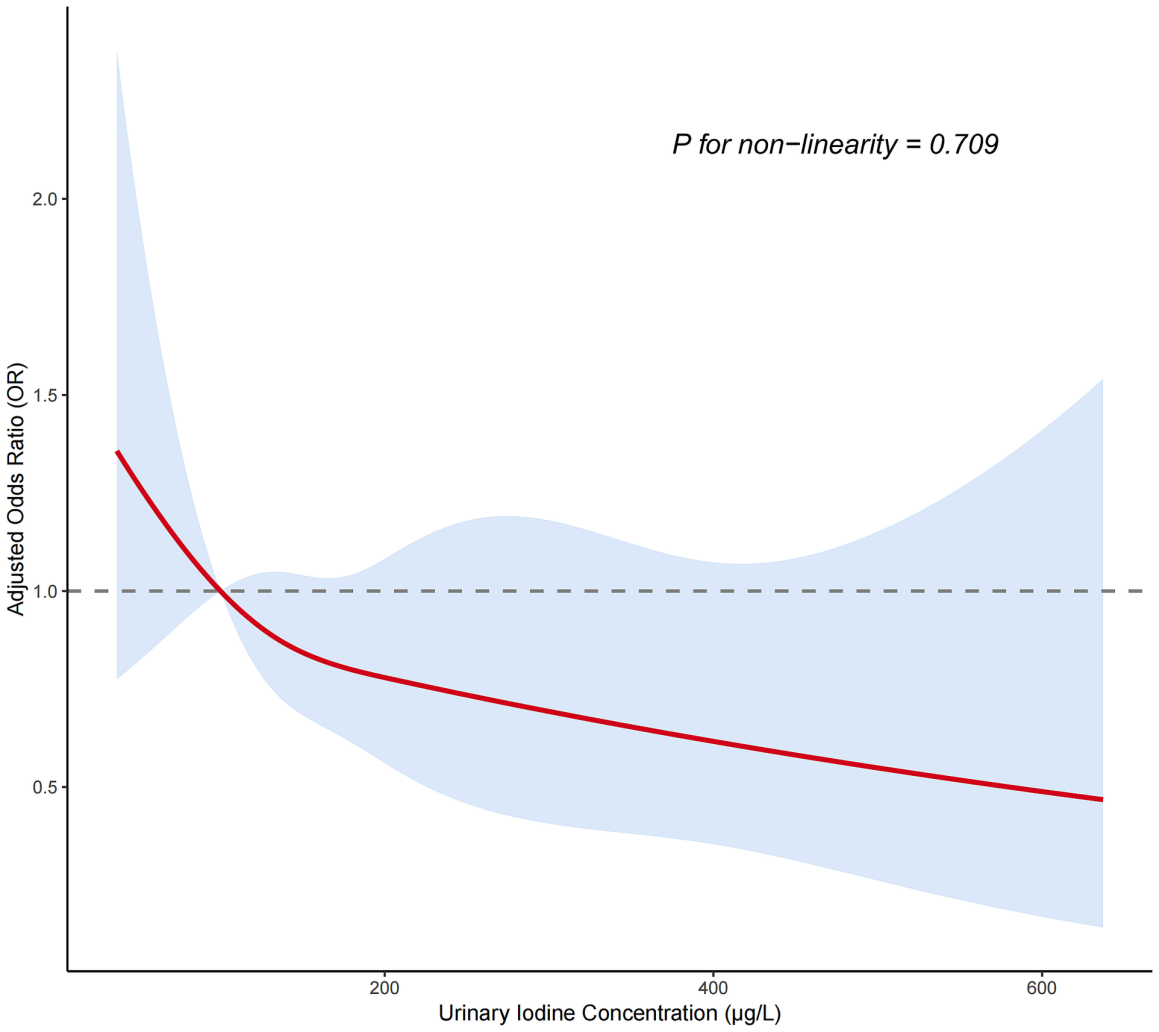
Dose–response association between UIC and the odds of LGS.

**Figure 4:**
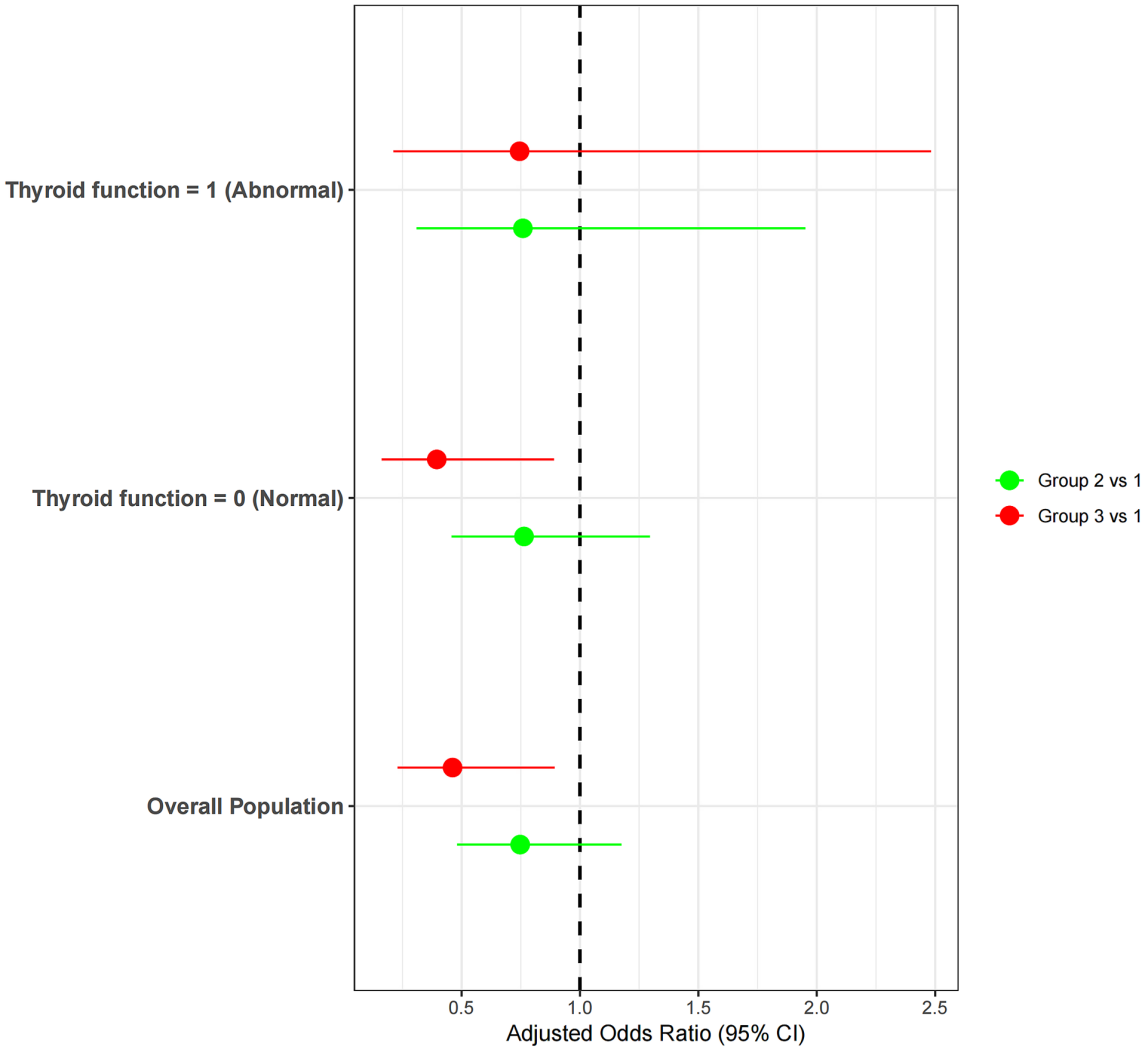
Association of UIC group with LGS stratified by thyroid function.

Trend analysis showed that the prevalence of LGS followed a U-shaped distribution across different UIC groups (Figure 2). In the overall population, the prevalence of LGS was highest in the iodine-deficient group (UIC < 100 μg/L), lowest in the 300–499 μg/L range, and rose slightly when UIC exceeded 500 μg/L. This trend was observed in both men and women, with a more pronounced effect in men (*p* < 0.05). We used RCS analysis to investigate the dose–response relationship between UIC and the odds of LGS. Three inflection points were selected based on Akaike Information Criterion (AIC) values, and UIC was modeled as a continuous variable. After adjusting for potential confounders, the results of the nonlinearity test were not significant (*P*_non-linear_ > 0.05). As UIC increased, the adjusted odds ratio (OR) remained consistently below 1.0, indicating that sustained iodine exposure was generally negatively associated with the probability of LGS (Figure 3). Stratification by thyroid function revealed that among participants with normal thyroid function, the odds of LGS were significantly lower in the groups with higher urinary iodine concentrations (Groups 2 and 3) compared to the group with the lowest urinary iodine concentration (Group 1). However, no statistically significant association was observed among participants with abnormal thyroid function (Figure 4).

### 3.3 Construction of the nomogram prediction model

**Figure 5:**
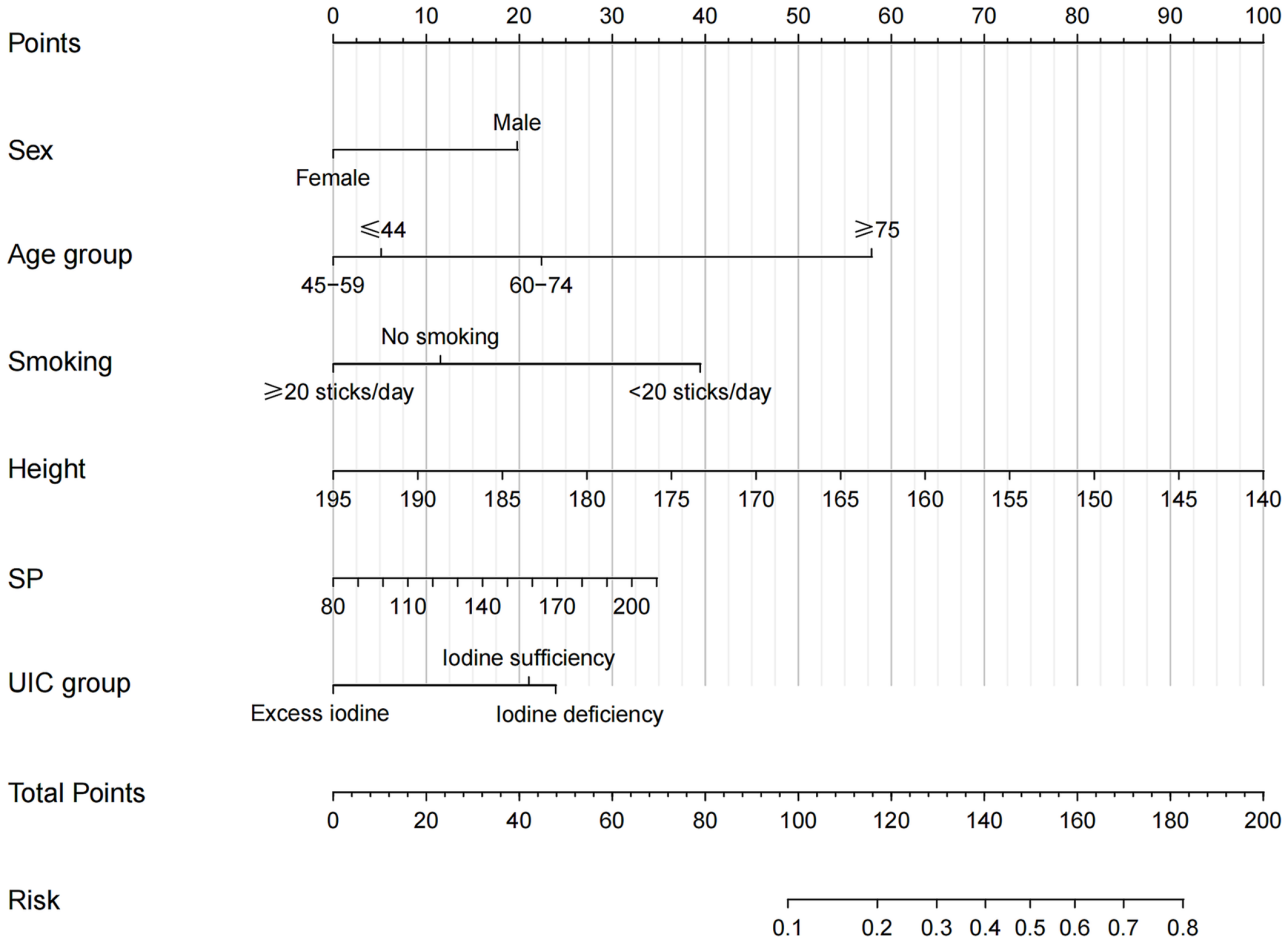
Nomogram prediction model for LGS.

The LGS was treated as the dependent variable (0 = No, 1 = Yes). Seven predictors that were significant in the univariate analysis [age group, smoking status, height, weight, FT4, UIC group, systolic blood pressure (SP)], together with literature-supported variables (sex and UA), were included in a multivariate logistic regression model. The Enter method was used to screen the variables with a test value of *α* = 0.05 to finalize the six influencing factors and ensure no severe multicollinearity (all VIFs < 5). Female sex (OR = 0.34, 95% CI: 0.17–0.69) and height (OR = 0.94, 95% CI: 0.89–0.98) were inversely associated with LGS. Excess iodine showed an inverse association with LGS (OR = 0.36, 95% CI: 0.14–0.86). Conversely, age (60–74 years: OR = 2.20, ≥75 years: OR = 9.73), smoking (OR = 3.20, 95% CI: 1.42–7.23), and SP (OR = 1.02, 95% CI: 1.01–1.03) were independently associated with increased odds of LGS (*p <* 0.05, Table 2). These variables were used to construct a nomogram prediction model (Figure 5).

**Table 2:** Results of the multi-variable logistic regression analysis.

Variables	*β*	S. E	*Z*	*P*	OR (95% CI)
Sex					
Male					
Female	−1.06	0.36	−2.93	0.003*	0.34 (0.17 ~ 0.69)
Age group					
1 (≤ 44)					
2 (45–59)	−0.13	0.29	−0.46	0.433	0.79 (0.44 ~ 1.45)
3 (60–74)	0.95	0.30	3.13	0.013*	2.20 (1.18 ~ 4.09)
4 (≥75)	2.46	0.59	4.18	<0.001*	9.73 (3.01 ~ 31.49)
Smoking					
No smoking					
<20 sticks/day	1.13	0.41	2.72	0.005*	3.20 (1.42 ~ 7.23)
>20 sticks/day	−0.47	0.37	−1.25	0.224	0.64 (0.36 ~ 1.32)
Thyroid function					
Normal					
Abnormal	−0.29	0.29	−0.99	0.324	0.75 (0.42 ~ 1.33)
Height	−0.07	0.02	−2.80	0.009*	0.94 (0.89 ~ 0.98)
Weight	−0.00	0.01	−0.16	0.442	0.99 (0.96 ~ 1.02)
FT4	−0.09	0.06	−1.54	0.086	0.90 (0.81 ~ 1.01)
UA	−0.00	0.00	−1.23	0.214	1.00 (1.00 ~ 1.00)
SP	0.015	0.007	4.13	0.042*	1.02 (1.01 ~ 1.03)
UIC group					
Iodine deficiency					
Iodine sufficiency	−0.12	0.27	−0.43	0.783	0.93 (0.55 ~ 1.60)
Excess iodine	−1.06	0.46	−2.28	0.026*	0.36 (0.14 ~ 0.86)

*P < 0.05.

### 3.4 Evaluation and validation of nomogram

**Figure 6:**
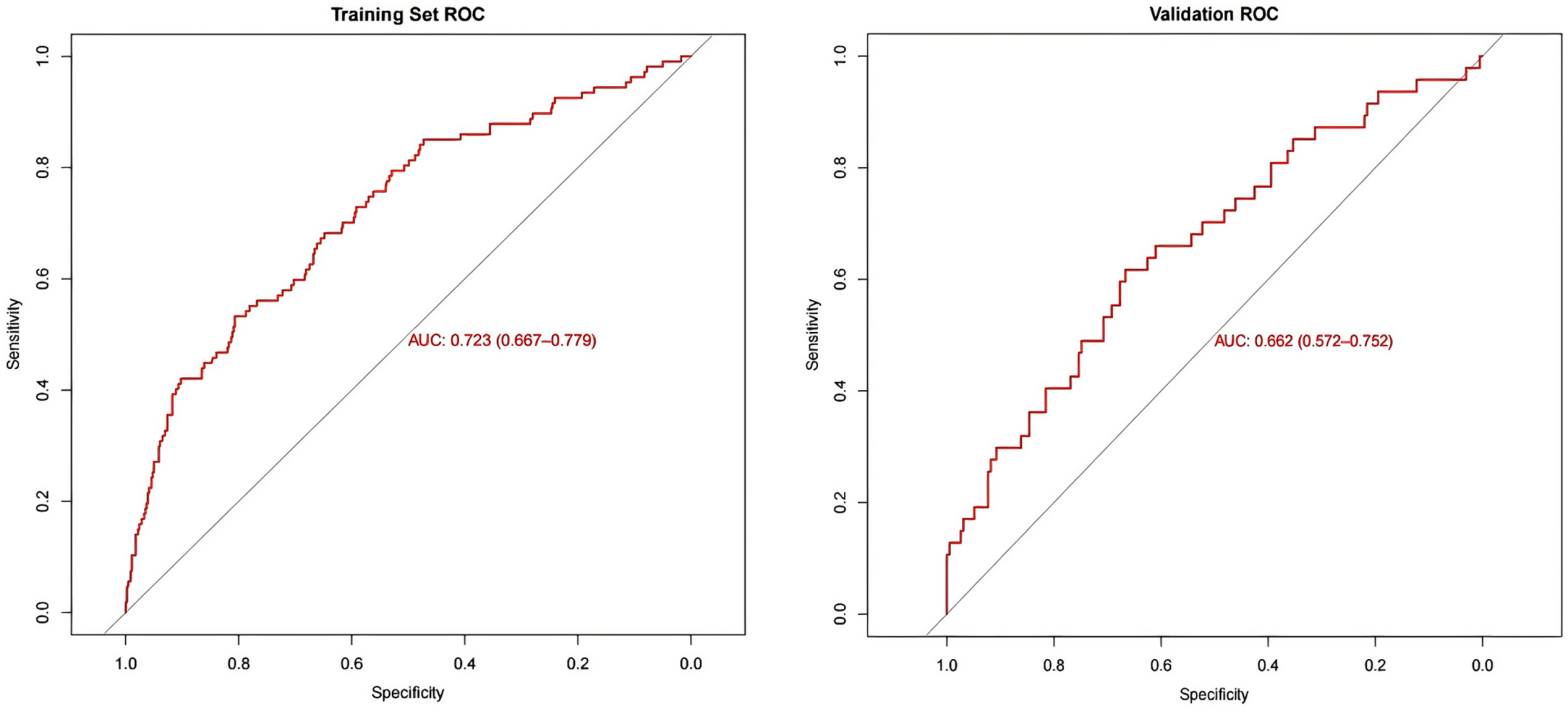
ROC curves of the nomogram.

**Figure 7:**
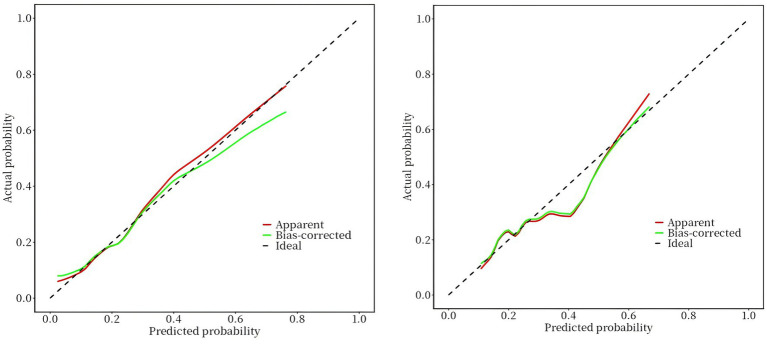
Calibration curves of the nomogram.

To evaluate the added predictive value of iodine status, we compared a basic clinical model (Model 1: sex, age group, smoking, height, and SP) with our extended nomogram model (Model 2: Model 1 and UIC group). The basic model achieved an AUC of 0.710 (95% CI: 0.655–0.765). Incorporating the UIC group improved the AUC to 0.723 (95% CI: 0.667–0.779) in the training set. The AUC value of the validation set was 0.662 (95% CI: 0.572–0.752), and the computational results revealed that the nomogram has moderate overall discrimination (Figure 6). The results of bootstrap method validation show that the calibration curve is in good agreement with the actual curve (training set: Brier score = 0.134, validation set: Brier score = 0.147). The Hosmer–Lemeshow test confirmed adequate calibration (training set: *χ*^2^ = 6.75, *p* = 0.56; validation set: *χ*^2^ = 12.11, *p* = 0.15) (Figure 7).

### 3.5 Decision curve analysis of the nomogram

**Figure 8:**
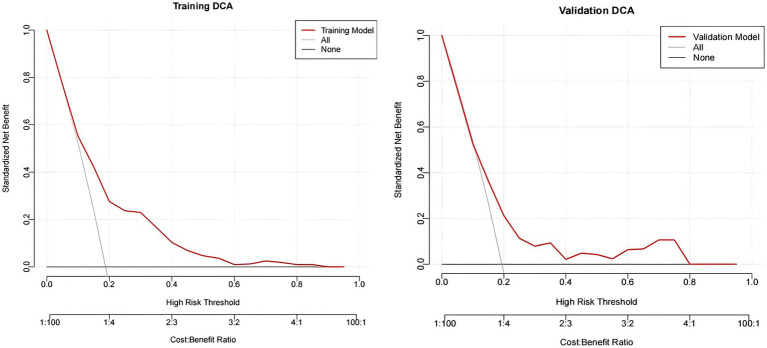
Decision curve of the nomogram.

The decision curve shows that when the threshold is within the range of 0.1–0.9, using the nomogram to inform clinical screening for LGS provides a higher net clinical benefit than either a “treat-all” or “treat-none” strategy (Figure 8).

## 4 Discussion

Sarcopenia and LGS impose significant physical and psychological burdens on the geriatric population. While traditional risk factors for muscle loss are well-documented, the role of trace elements such as iodine remains underexplored, hindering early clinical detection. This study developed a prediction nomogram integrating UIC with routine clinical variables. In contrast to machine learning-based predictive models that often require complex measurement equipment (11), our nomogram relies solely on readily available clinical data, offering a cost-effective and accessible tool for risk assessment. By providing a quantitative, objective, and cost-effective risk assessment, this tool facilitates the early identification of at-risk individuals in primary care settings.

Five independent factors were identified as significant correlates of LGS probability. They include female sex, older age, shorter height, smoking, and higher SP. Sex differences in LGS likely result from hormonal regulation. These findings align with previous epidemiological observations reported by Ribeiro et al. (12). Men have higher muscle mass related to testosterone. This hormone declines with age by approximately 1.2% per year in Asian men (13), accelerating muscle loss. Postmenopausal estrogen deficiency may also impair muscle function. Occupational and physical activity patterns contribute further. Men more often engage in high-intensity labor, potentially causing chronic muscle strain (14, 15). Age is another major factor for LGS. Physical function naturally drops as people get older (16). Specifically, grip strength drops much faster after people reach 50 years old (17). Sarcopenia risk demonstrates a significant age-dependent progression. Each decade of aging increases the risk of muscle loss by 1.68 times (18). The human body goes through many declines with aging. Older people have poorer stomach absorption and more diseases. Their muscle fibers shrink, and their nerve-muscle links weaken. All these problems lead to progressive muscle weakness (19). Taller people have a lower probability of LGS (OR = 0.929). McGovern et al. previously confirmed this direct link between stature and muscle function. They found that every 1 cm increase in height boosts grip strength by about 0.026 standard deviations in men and 0.018 in women (20). This structural advantage likely originates from early human development. Skeletal mechanisms, such as endochondral ossification, directly build a larger muscle framework. Gene regulatory pathways also connect early physical growth to late-life muscle capacity (21). Smokers have a much higher risk of LGS. This finding matches previous long-term studies (22, 23). Smoking narrows blood vessels and damages their inner walls. Nicotine also causes oxidative stress. This stress breaks muscle cell membranes, harms nerve-muscle links, and speeds up protein breakdown and ruins muscle strength. There was a significant inverse correlation between systolic blood pressure and grip strength; each 1 mmHg increase in SP was associated with higher odds of LGS (OR = 1.02, 95% CI: 1.01–1.03). These findings are consistent with prior large-scale studies. Kwon et al. (24) reported that higher SP was inversely associated with dominant-hand grip strength in a nationwide Korean cohort. Cross-sectional analyses in other populations, including physically disabled older adults in China (25) and community-dwelling cohorts (26), also observed similar inverse associations. Hypertension may contribute to muscle weakness through vascular damage. Chronic high blood pressure promotes microvascular injury and arterial stiffness, reducing blood flow and oxygen delivery to skeletal muscle, which impairs nutrient supply and protein synthesis, ultimately accelerating sarcopenia and functional decline.

We observed UIC as an independent factor associated with LGS, showing a U-shaped relationship in unadjusted analyses. This pattern is consistent with prior research linking urinary iodine to metabolic disorders (27) and all-cause mortality (28). Biologically, this association may operate through two interrelated mechanisms. Iodine directly affects cellular energy metabolism by facilitating glucose uptake into adipocytes, thereby providing energy for muscle contraction (29). Recent studies support this mechanism, reporting positive correlations between urinary iodine and muscle mass in young adults (30, 31). Iodine also acts indirectly via thyroid hormone synthesis. Adequate iodine is essential for thyroid hormone production, which regulates muscle fiber development and neuromuscular function (32). A systematic review confirms that these thyroid hormones play a major role in maintaining overall muscle mass and strength (33). The subgroup analysis supports this thyroid-mediated effect, and the protective association of UIC was strongest among euthyroid participants (34, 35). The U-shaped relationship warrants careful interpretation. Moderate iodine levels appear beneficial, but the association in the excess iodine group was statistically imprecise, with an odds ratio of 0.36 and a wide confidence interval. This statistical imprecision is primarily attributable to the small sample size in the excess iodine group (*n* = 79). Furthermore, studies have shown that Gansu Province was previously an iodine-deficient region (36). The study sample had a highly skewed UIC distribution, with very few participants exceeding 500 μg/L, which further limited the ability to observe the relationship between high iodine intake and grip strength. Previous studies have shown that chronic excessive iodine intake impairs pancreatic β-cell function (29) and disrupts thyroid homeostasis (37, 38). These toxic effects potentially offset any muscular benefit. These findings highlight that the relationship between iodine range and muscle health is not a simple linear dose-response relationship. From a public health perspective, the findings offer pragmatic insights for mitigating age-related muscle decline. Integrating routine community-based iodine monitoring with targeted resistance training programs could represent a highly effective, dual-action intervention strategy for vulnerable older populations. Thyroid hormones clearly act as a primary mediator, but many studies suggest that the gut–thyroid axis may also play a role in maintaining musculoskeletal health (39). The gut microbiome affects both iodine absorption and thyroid hormone metabolism by modulating the bioavailability of key micronutrients such as selenium and zinc (40). Because skeletal muscle depends on thyroid hormone signaling, disruptions in this gut-endocrine network may contribute to age-related muscle loss. Future studies should further investigate this complex neuroendocrine-microbiome interaction.

This study has several limitations. First, because this study employed a cross-sectional design, it is not possible to establish a causal relationship, nor can reverse causality be ruled out (for example, patients who experience early-onset muscle weakness may alter their dietary habits, thereby affecting their iodine intake and leading to changes in urinary iodine concentration). While we identified an association between UIC and LGS, further research is needed to explore the underlying complex mechanisms. Second, urine samples were collected only once from each participant. Although participants with specific recent dietary or medication intake were excluded, daily fluctuations in individual iodine levels may have influenced the study results. Future studies could collect more stable indicators, such as serum iodine or salivary iodine, to assess individual iodine nutritional status. Third, as Gansu Province has a history of iodine deficiency, the sample for this study was exclusively drawn from this region. This resulted in a small excess-iodine subgroup, which, along with the single-province design, limits the generalizability of the research results. In addition, several key confounding factors were not assessed, including individual psychosocial factors [such as depression and loneliness (41, 42)], detailed dietary sources of iodine, levels of physical activity, and comorbidities (such as diabetes and vitamin D status). Future research should comprehensively collect both physiological and psychological data for integrated analysis, while conducting longitudinal studies and multicenter external validation to expand the model’s clinical applicability. Finally, the UIC and LGS-specific molecular mechanisms remain unclear. Future animal and cellular studies are needed to explore these mechanisms.

## 5 Conclusion

There was developed a predictive nomogram for LGS based on UIC and routine clinical variables. The nomogram demonstrated moderate discriminative ability and acceptable calibration in our internal validation, suggesting its potential utility as a screening tool for LGS in community-dwelling older adults, helping clinicians identify individuals at higher risk and guide personalized interventions, such as nutrition or resistance training. However, this nomogram should be viewed as a preliminary community screening aid rather than a definitive diagnostic tool. Future studies should include larger, multi-center populations and explore whether iodine dysregulation directly contributes to LGS through functional experiments, thereby providing stronger evidence for the prevention and clinical management of LGS.

## Data Availability

The raw data supporting the conclusions of this article will be made available by the authors, without undue reservation.
