# Supplementary material for: Relationship between iodine nutritional status and low handgrip strength in a Northwestern Chinese cohort: construction of a predictive nomogram model
Source: Front Nutr. 2026 Apr 14;13:1793869. doi: 10.3389/fnut.2026.1793869 (PMC13120904; doi:10.3389/fnut.2026.1793869)
Supplement: Supplementary file 1 [file Supplementary_file_1.docx]

**Table S1 Model coefficients, 95% confidence intervals (CIs), and variance inflation factors (VIFs) for the multivariable logistic regression model.**

| **Variables** | ***β*** | **S.E** | ***β* (95% CI)** | **VIF*** |
| --- | --- | --- | --- | --- |
| **Sex** |  |  |  | 2.21 |
| Male |  |  |  |  |
| Female | -0.834 | 0.340 | -1.51 - 0.170 |  |
| **Age group** |  |  |  | 1.08 |
| 1 (≤ 44) |  |  |  |  |
| 2 (45-59) | -0.217 | 0.290 | -0.794 - 0.347 |  |
| 3 (60-74) | 0.728 | 0.310 | 0.114 - 1.330 |  |
| 4 (≥75) | 2.220 | 0.583 | 1.100 - 3.420 |  |
| **Smoking** |  |  |  | 1.16 |
| No smoking |  |  |  |  |
| <20sticks/day | 1.180 | 0.409 | 0.359 - 1.970 |  |
| >20sticks/day | -0.486 | 0.370 | -1.240 - 0.220 |  |
| **Height** | -0.077 | 0.022 | -0.120 -0.035 | 2.09 |
| **SP** | 0.011 | 0.007 | -0.003 - 0.025 | 1.11 |
| **UIC group** |  |  |  | 1.02 |
| Iodine deficiency |  |  |  |  |
| Iodine sufficiency | -0.122 | 0.265 | -0.634 - 0.408 |  |
| Excess iodine | -1.010 | 0.457 | -1.960 -0.155 |  |

***:** The coefficients (*β*) and their standard errors (S.E.) were derived from the multivariable logistic regression model. For multi-categorical variables (Age group, Smoking status, and UIC group), the Generalized Variance Inflation Factor (GVIF) was calculated. To ensure comparability with the traditional VIF threshold (VIF < 5 indicates no severe multicollinearity), the reported VIF values for multi-categorical variables were transformed using the formula: (GVIF^1/(2*Df)^)^2^.
