# Supplementary material for: Relationship between iodine nutritional status and low handgrip strength in a Northwestern Chinese cohort: construction of a predictive nomogram model
Source: Front Nutr. 2026 Apr 14;13:1793869. doi: 10.3389/fnut.2026.1793869 (PMC13120904; doi:10.3389/fnut.2026.1793869)
Supplement: Supplementary file 2 [file Supplementary_file_2.docx]

# Supplementary Script: Nomogram Construction and Validation

=============================================================

# 1. Initialization and Package Loading

=============================================================

rm(list = ls())

# Load required packages

library(rms) # For logistic regression and nomogram

library(pROC) # For ROC curve analysis

library(rmda) # For Decision Curve Analysis (DCA)

library(dplyr) # For data manipulation

library(ResourceSelection) # For Hosmer-Lemeshow test

# =============================================================

# 2. Data Import and Preprocessing

=============================================================

# Note: Users should replace "path_to_train.csv" and "path_to_test.csv"

# with their actual local file paths.

train_data <- read.csv(file.choose())

test_data <- read.csv(file.choose())

preprocess_data <- function(df) {

# Convert target variable to numeric (0 or 1)

df$LGS <- as.numeric(as.character(df$LGS))

# Factorize categorical variables with proper labels

df <- df %>% mutate(

Sex = factor(Sex, levels = c(1, 2), labels = c("Male", "Female")),

Age_group = factor(Age_group, levels = c(1, 2, 3, 4), labels = c("≤ 44", "45-59", "60-74", "≥75")),

Smoking = factor(Smoking, levels = c(1, 2, 3), labels = c("No smoking", "<20 sticks/day", "≥20 sticks/day")),

UIC_group = factor(UIC_group, levels = c(1, 2, 3), labels = c("Iodine deficiency", "Iodine sufficiency", "Excess iodine"))

)

return(df)

}

train_data <- preprocess_data(train_data)

test_data <- preprocess_data(test_data)

=============================================================

# 3. Model Construction and Nomogram (Training Set)

=============================================================

# Set data distribution for rms package

ddist_train <- datadist(train_data)

options(datadist = "ddist_train")

# Fit the multivariable logistic regression model

model <- lrm(LGS ~ Sex + Age_group + Smoking + Height + SP + UIC_group,

data = train_data, x = TRUE, y = TRUE)

print(model)

# Generate and plot the nomogram

nom <- nomogram(model,

fun = plogis,

funlabel = "Risk",

lp = FALSE,

abbrev = FALSE)

plot(nom, cex.var = 0.9, cex.axis = 0.8, col.grid = gray(c(0.8, 0.95)))

# =============================================================

# 4. Model Evaluation: Training Set

=============================================================

train_data$pred <- predict(model, type = "fitted")

# 4.1 ROC Curve

roc_train <- roc(train_data$LGS, train_data$pred, ci = TRUE)

plot(roc_train, print.auc = TRUE, auc.polygon = FALSE,

main = "Training Set ROC", col = "red", lwd = 2)

# 4.2 Brier Score and Hosmer-Lemeshow Test

brier_train <- mean((train_data$pred - train_data$LGS)^2)

hl_train <- hoslem.test(train_data$LGS, train_data$pred, g = 10)

cat(sprintf("Training Brier Score: %.4f\n", brier_train))

print(hl_train)

# 4.3 Calibration Curve (Matching the requested aesthetic style)

cal_train <- calibrate(model, method = "boot", B = 1000)

plot(0, 0, type = "n", xlim = c(0, 1), ylim = c(0, 1),

xlab = "Predicted probability", ylab = "Actual probability")

abline(0, 1, col = "black", lty = 2, lwd = 2) # Ideal line

lines(cal_train[, "predy"], cal_train[, "calibrated.orig"], type = "l", lwd = 2, col = "red") # Apparent

lines(cal_train[, "predy"], cal_train[, "calibrated.corrected"], type = "l", lwd = 2, col = "#00FF00") # Bias-corrected

legend("bottomright", legend = c("Apparent", "Bias-corrected", "Ideal"),

lty = c(1, 1, 2), lwd = c(2, 2, 2), col = c("red", "#00FF00", "black"), bty = "n")

# 4.4 Decision Curve Analysis (DCA)

dca_train <- decision_curve(LGS ~ pred, data = train_data, fitted.risk = TRUE,

thresholds = seq(0, 1.0, by = 0.05), confidence.intervals = FALSE)

plot_decision_curve(dca_train, curve.names = "Training Model", col = "red",

xlim = c(0, 1.0), ylim = c(0, 1), main = "Training DCA")

# =============================================================

# 5. Model Evaluation: Internal Validation Set

=============================================================

# Predict probabilities on the external/internal validation set

test_data$pred <- plogis(predict(model, newdata = test_data))

# 5.1 ROC Curve

roc_test <- roc(test_data$LGS, test_data$pred, ci = TRUE)

plot(roc_test, print.auc = TRUE, auc.polygon = FALSE,

main = "Validation Set ROC", col = "red", lwd = 2)

# 5.2 Brier Score and Hosmer-Lemeshow Test

brier_test <- mean((test_data$pred - test_data$LGS)^2)

hl_test <- hoslem.test(test_data$LGS, test_data$pred, g = 10)

cat(sprintf("Validation Brier Score: %.4f\n", brier_test))

print(hl_test)

# 5.3 Calibration Curve (Matching the requested aesthetic style)

plot(0, 0, type = "n", xlim = c(0, 1), ylim = c(0, 1),

xlab = "Predicted probability", ylab = "Actual probability")

abline(0, 1, col = "black", lty = 2, lwd = 2) # Ideal line

# Calculate Apparent (Loess) curve for validation

loess_fit_test <- loess(LGS ~ pred, data = test_data, span = 0.75, degree = 2)

p_seq_test <- seq(min(test_data$pred, na.rm=TRUE), max(test_data$pred, na.rm=TRUE), length.out = 100)

cal_apparent <- predict(loess_fit_test, newdata = data.frame(pred = p_seq_test))

lines(p_seq_test, cal_apparent, type = "l", lwd = 2, col = "red")

# Calculate Bias-corrected (Logistic Calibration) curve for validation

logit_pred <- qlogis(test_data$pred)

logistic_fit <- glm(LGS ~ logit_pred, family = binomial, data = test_data)

cal_corrected <- predict(logistic_fit, newdata = data.frame(logit_pred = qlogis(p_seq_test)), type = "response")

lines(p_seq_test, cal_corrected, type = "l", lwd = 2, col = "#00FF00")

legend("bottomright", legend = c("Apparent", "Bias-corrected", "Ideal"),

lty = c(1, 1, 2), lwd = c(2, 2, 2), col = c("red", "#00FF00", "black"), bty = "n")

# 5.4 Decision Curve Analysis (DCA)

dca_test <- decision_curve(LGS ~ pred, data = test_data, fitted.risk = TRUE,

thresholds = seq(0, 1.0, by = 0.05), confidence.intervals = FALSE)

plot_decision_curve(dca_test, curve.names = "Validation Model", col = "red",

xlim = c(0, 1.0), ylim = c(0, 1), main = "Validation DCA")

=============================================================

# End of Script

=============================================================
